# Effect of Mobile-health on maternal health care service utilization in Eastern Ethiopia: study protocol for a randomized controlled trial

**DOI:** 10.1186/s13063-018-2446-5

**Published:** 2018-02-12

**Authors:** Tilayie Feto Gelano, Nega Assefa, Yadeta Dessie Bacha, Afendi Abdi Mahamed, Kedir Teji Roba, Mitiku Teshome Hambisa

**Affiliations:** 1 Department of Pediatrics and Neonatal Nursing, School of Nursing and Midwifery, College of Health and Medical Sciences, Haramaya University, Harar, PO Box 235, Ethiopia; 2 School of Nursing and Midwifery, Department of Public Health, College of Health and Medical Sciences, Haramaya University, Harar, Ethiopia; 3 School of Public Health, College of Health and Medical Sciences, Haramaya University, Harar, Ethiopia; 4 Department of Computer and Information Sciences, College of Sciences and Technology, Haramaya University, Harar, Ethiopia; 5 Department of Nursing, School of Nursing and Midwifery, College of Health and Medical Sciences, Haramaya University, Harar, Ethiopia; 6 Research Centre for Generational Health and Ageing, University of Newcastle, P.O. Box 2308, Callghan, New Castle, NSW Australia

**Keywords:** Mobile-health, Maternal health, Eastern Ethiopia, Randomized controlled trial

## Abstract

**Background:**

Globally, the rapid development of mobile technology has created new ways of addressing public health challenges and shifted the paradigm of health care access and delivery. The primary aim of this study is to examine the effectiveness of Mobile-health on maternal health care service utilization in Eastern Ethiopia.

**Methods/design:**

Through, a cluster-randomized controlled trial, 640 participants will be selected based on their districts and respective health centers as the unit of randomization. All pregnant mothers who fulfill the inclusion criteria will be allocated to a mobile-phone-based intervention and existing standard of care or control with a 1:1 allocation ratio. The intervention consists of a series of 24 voice messages which will be sent every 2 weeks from the date of enrollment until the close-out time. The control group will receive existing standard of care without voice messages. Data related to outcome variables will be assessed at three phases of the data collection periods. The primary outcome measures will be the proportion of antenatal care visits and institutional delivery, whereas the secondary outcome measures will consist of the proportion of postnatal care visits and pregnancy outcomes. Risk ratios will be used to a measure the effect of intervention on the outcomes which will be estimated with 95% confidence interval and all the analyses will be done with consideration of clustering effect.

**Discussions:**

This study should generate evidence on the effectiveness of mobile-phone-based voice messages for the early initiation of maternal health care service use and its uptake. It has been carefully designed with the assumption of obtaining higher levels of maternal health care service use among the treatment group as compared to the control.

**Trial registration:**

Pan African Clinical Trial Registry, www.panctr.org, ID: PACTR201704002216259. Registered on 28 April 2017.

**Electronic supplementary material:**

The online version of this article (10.1186/s13063-018-2446-5) contains supplementary material, which is available to authorized users.

## Background

Mobile phones, which have reached the hands of 90% of the world’s population, of whom 80% are rural dwellers [1], has also made possible Mobile-health/m-health, the usage of mobile technology to improve health. Globally, the rapid development of mobile technology has generated new ways to address public health challenges and shifted the paradigm of health care access and delivery [1, 2]. In the health care system of developing countries, mobile-phone technology is used to report health information and health care is delivered through telemedicine, to populations otherwise deprived, to improve adherence to treatment and appointments [3]. m-health also plays a major role in sexual and reproductive health care and is seen as a key area in achieving the global strategy for women’s and children’s health [4]. Evidence has shown that m-health minimizes time barriers and facilitates urgent care during emergency obstetric referrals and maternal health care use in low- and middle-income countries (LMICs) [5].

Developing regions accounted for approximately 99% of global maternal deaths in 2015, of which sub-Saharan Africa (SSA) alone accounted for about two thirds, followed by one third in Southern Asia. Achieving the Sustainable Development Goals (SDG) target of a maternal mortality rate (MMR) below 70 per 100,000 live births will require a 7.5% reduction of MMR every year between 2016 and 2030 [6]. This will need more than three times the annual rate of global maternal mortality reduction that was observed between 1990 and 2015 [7]. Furthermore, three million babies die every year before they are a month old, and a similar number are stillborn [8, 9]. Most of the deaths are due to lack of quality maternal and neonatal health care service provision [10].

At the moment, many countries, including Ethiopia, are working to meet the recommendation of having skilled birth attendants for all births [11]. However, the maternal health problem is still very serious; every year about 350,000 maternal deaths occur because of pregnancy and its related complications. The problem is more serious in developing countries [11, 12]. In Ethiopia, the mortality rate increased from 673 per 100,000 live births in 2005 to 676 per 100,000 live births in 2011 [13].

Since 2003, Ethiopia has had increasing access to primary health care services through the Community Based Health Extension Program (CBHEP). Between 2003 and 2010, about 34,000 health extension workers (HEWs) were trained and deployed in 15,000 kebeles (villages), each of which had one newly constructed health post (HP) and about 3000–5000 dwellers [12]. Regardless of all these efforts, according to the Ethiopian Demographics Health Survey (EDHS) 2011, the number of women who received antenatal care (ANC) (34%), institutional delivery (10%) and postnatal care (PNC) (6%) services by skilled workers was very small, and this has resulted in high maternal and neonatal morbidity and mortality [11]. To tackle this problem more effectively, practicing innovative ways of health care service delivery, which will be integrated with the existing health care system is crucial, and thus mobile technology is being used. Despite the widespread use of mobile technologies in the health care system, there is limited evidence on the efficacy of mobile-phone-based interventions, or m-health, on maternal health care service utilization. In Ethiopia, no study has been conducted on the topic. To fill the gap, this study, through a randomized controlled trial (RCT), examines the effects of m-health on maternal health care service use in Eastern Ethiopia. It is hypothesized that m-health will increase maternal health care service provision in limited-resource and -literacy settings. In this RCT, two districts that are 34 km apart will be selected to reduce the possible risk of contamination between the intervention and control arms and to evaluate the barriers of implementation. The outcomes of this investigation will be analyzed for both intervention and control arms separately. Examining challenges and outcomes at this stage will provide insight into m-health implementation for improved maternal health care service use.

## Methods/design

### Study design

**Fig. 1:**
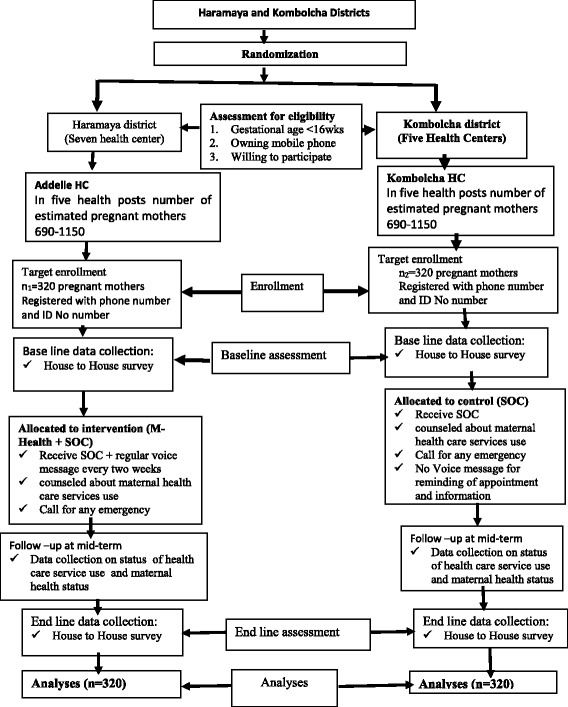
Consolidated Standards of Reporting Trials (CONSORT) Diagram of study design. This figure depicts sampling method and allocation of study units, assessment of eligibility criteria to include study participants

A single-blind, cluster-RCT study design will be employed on districts and their respective health centers (HCs). Study participants will be randomized to a mobile-phone-based intervention (voice message for reminding and informing about the advantages of maternal health care service use) or existing standard of care (SOC)/control (without voice message) under their respective districts and HCs with a 1:1 allocation ratio (Fig. 1).

**Fig. 2:**
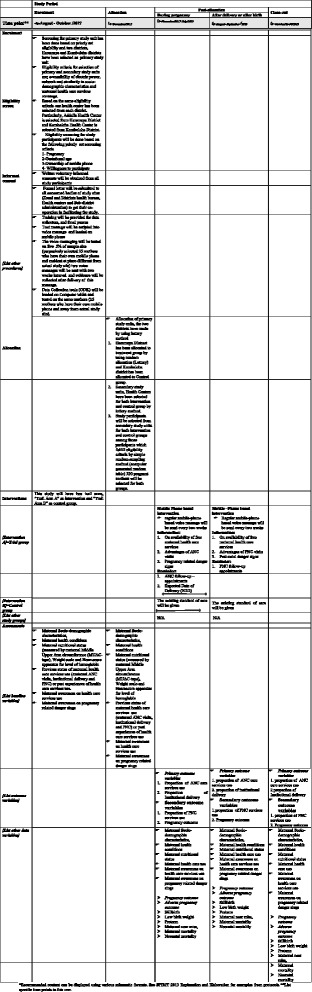
Standard Protocol Items: Recommendations for Interventional Trials (Additional file 1: SPIRIT) Figure for the schedule of enrollment, interventions and assessments. This figure shows the schedule of enrollment, interventions and assessments of the m-health trial study, Eastern Ethiopia, August 2017 to October 2018

**Fig. 3:**
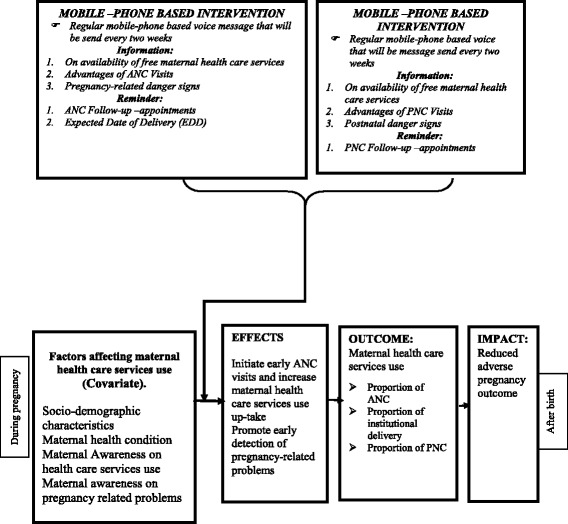
Summary of conceptual framework. This figure shows that the effects of mobile-phone-based voice message on maternal health care service use

### Study setting

This study will be done in Haramaya and Kombolcha districts, which are in Eastern Haaraghe Zone, Oromia Regional State, Eastern Ethiopia.

Haramaya district, which is about 506 km from Addis Ababa, the capital city of Ethiopia, is located between 9°24′N and 42°01′E and is 1400–2340 m above sea level. According to the 2007 national census of the country, its population was 271,018, of whom 48.97% were women and 18.46% were urban dwellers. Moreover, by using pregnancy rate estimation factors of 4.6%, around 12,467 women are expected to be pregnant in this district. In the district, there are seven HCs, 22 HPs and one hospital, [14].

On the other hand, the district of Kombolcha is 540 km from Addis Ababa and is found at between 1200 and 2460 m above sea level. According to the 2007 national census report, its population was about 140,080, of whom 49.3% were women and 9.01% were urban dwellers. Moreover, by using pregnancy estimation factors of 4.6%, about 6444 women are expected to be pregnant in this district. In Kombolcha, there are five HCs and 22 HPs.

Related to study parameters, like socio-demographics, economic characteristics and maternal health care service coverage, the two districts have similarities [15].

### Participants: inclusion and exclusion criteria

The participants will be recruited from two districts (Haramaya and Kombolcha districts) based on pre-set eligibility criteria (pregnancy, gestational age (GA), ownership of a mobile phone and willingness to participate). The participant will be included in the trial if her GA is less than 16 weeks, she owns a mobile phone and has willingness to participate. The potential trial participants will be identified by the field worker-based enrollment criteria. For all eligible participants, the field workers will explain about study by reading the participant information sheets; if the study subjects are willing to participate, written informed voluntary consent will be obtained and baseline data collection will be made.

In this trial, study women who fulfill the enrollment criteria for the study but are critically ill at time of enrollment will be excluded from the study. Critically ill women are those pregnant mothers who unable to respond to interview questions because of illness. In addition, women with any systemic illness during this time will be excluded from the study. Any pregnant women with systemic illness need an special ANC follow- up, which is different from the recommended routine ANC follow -up and this set precondition without effect of treament for good adherence to maternal health care service use. Hence, any pregnant mother with known systemic illnesswill be excluded from this study.

### Sample size determination

A study which was conducted in Zanzibar showed that 60% of the intervention group used a health-professional-assisted delivery (institutional delivery), as compared to 47% in the control group; based on this evidence, sample size required for this study is calculated as follows [16]:

- P_1_ = proportion of health professional assisted delivery in control group = 47%
- P_2_ = proportion of health professional assisted delivery in intervention group = 60%

Based on the above information, a sample size required for this study is calculated by using STATA/SE software with consideration of sample size for a two-sample comparison of proportions [17].

### Assumption and hypothesis for sample size calculation

*H*_o_: *P*_1_ = *P*_2_, whereas *P*_1_ is the proportion in the intervention group (population _1_) and *P*_2_ is the proportion in the control group (population _2_), *α* is level of significance at 95% confidence interval, and *β* is power.

In a cluster-RCT the study subjects within the cluster are more likely to interact, respond in the same manner and can no longer act independently which can lead to a loss of statistical power. This intracluster dependence can be quantified by considering intracluster correction coefficient (ICC) and design effects (DE) [18]. Hence, the estimated sample size per group is inflated by considering ICC, and DE of 1.3. Thus, the final sample size required for this study will be 320 for each control and treatment groups.

*Description of sample size calculation*: a sample size of 640 pregnant mothers (320 in each of the treatment and control group) will be sufficient to detect a clinically important difference of 13% between groups in the m-health intervention, using a two-sided test of 80% power (*β*) and 5% of significance level (*α*) with 95% confidence interval**.** The 13% difference represents the difference between a 60% health-professional-assisted delivery rate in the intervention group as compared to the control group [16].

### Randomization and sampling procedure

Among the 15 districts in Eastern Hararghe Zone, Haramaya and Kombolcha are selected purposefully because they have better access to electric power and network, which are mandatory for mobile-phone charging and communication during the study period. In addition, the community in these districts has similar socioeconomic and demographic characteristics and maternal health care service coverage [19, 20]. Then, by a lottery method, the two districts are determined as an intervention (m-health + SOC) or a control group (SOC only). Accordingly, Haramaya district is an intervention group (m-health + SOC) whereas Kombolcha is a control (SOC).

Based on the EHSTP of 2016–2020, one HC, which encompasses five HPs in five Kebeles which consisting of 3000–5000 people each, can provide service for 15,000–25,000 people [21]**.** In Ethiopia, the estimated number of pregnancies in a given population is about 4.6% [22]. Hence, the total number of estimated pregnant mothers in five kebeles surrounding one HC is about 690–1150.

Among the seven HCs in Haramaya district, Addelle HC is selected by the purposive method as secondary study unit. The main reason for the purposive selection is accessibility of electric power and network, which will be mandatory for mobile-phone charging and communication during the study period. Finally, all pregnant mothers who fulfill the enrollment criteria in the selected Kebele will be registered and 320 pregnant mothers will be selected for the intervention group by a simple random-sampling method. Likewise, of the five HCs in Kombolcha district, Kombolcha HC is selected purposefully for the same reason, and from the surrounding kebeles, 320 mothers will be selected for the control group by simple random sampling (SRS) method.

#### Screening and enrollment procedure

In this study, pregnancy, GA and ownership of mobile phone will be used as the main selection and enrollment criteria. Hence, in the selected kebeles, first, all the reproductive-age women with a history of amenorrhea for the last 28 days, who do not use any family planning, and who own a mobile phone will be screened for pregnancy through beta-human chorionic gonadotropin (HCG) urine test. Then, the mothers who are found to be positive for the HCG urine test will be given a unique identification number (ID No) and screened for GA through their last menstrual period (LMP) and/or portable ultrasound (U/S) scan result. Next, those women of less than 16 weeks of GA will be registered as illegible for the study. Finally, for each control and intervention group, 320 pregnant women who fulfill the enrollment criteria will be selected by a simple random-sampling method (computer-generated random table) [23]. They will be registered with their ID No and mobile phone number on a computer tablet.

### Data collection method and tools

Data will be collected in three phases (phases I, II and III) using a questionnaire loaded on four computer tablets, and by four trained BSc nurses and six health extension workers (HEWs).

#### Phase I

In this phase, for both intervention and control groups, a baseline survey will be conducted on sociodemographic characteristics and maternal health care service use via a structured, interview-based questionnaire which will be adopted from similar studies. Maternal health status will be assessed through a medical interview checklist and nutritional status will be screened by using a mid upper-arm circumference (MUAC) tape, a weighing scale and a Hemacue machine or portable hemoglobin analyzers to test for hemoglobin level.

#### Phase II

This stage of data collection will be conducted after the eighth voice message or the fourth month of enrollment and the data collection will focus on maternal health condition, nutritional status and status of the voice messages which will be delivered to the intervention group, status of maternal health care service use among both intervention and control groups. During this period, data related to maternal health conditions will be collected through the same tools which will be used in phase I. In addition, data related to maternal health care services using status and voice message will be collected through a structured, interview-based questionnaire with confirmation of voice messages from their mobile phone.

#### Phase III

This phase of data collection will be done at study close-out and will be conducted after 24 voice messages or 45 days post delivery for every mother in the intervention and control groups. It will focus on maternal health condition, nutritional status, health care service use, pregnacy outcome and the status of the mobile-phone-based voice message since their enrollment time. Furthermore, mothers in the intervention group will be asked how voice messages helped them.

#### Intervention and follow-up procedures

This study will have two trial arms, “Trial arm A” is the intervention and “Trial arm B” is the control group.

#### Trial arm A

In this arm, a mobile-phone-based voice message consisting of information about the advantage of maternal health care service use (advantage of ANC, PNC and institutional delivery) and reminders of their appointment will be sent. Particularly, every 2 weeks a voice message composed of reminders for ANC follow-up and information about its advantages (first to fourth ANC visits) and expected date of delivery (EDD) will be sent. After birth, reminders for PNC follow-up (first to third PNC follow-up) and information about its advantages will be sent as voice messages through the mobile phone.

Published evidence supports the notion that the first ANC visit should be as early as possible in pregnancy, preferably in the first trimester (within the first 12 weeks of gestation) and in a normal pregnancy the protocol for pregnancy care recommends four ANC visits [24]. Hence, appointments for ANC follow-up will be adjusted based on GA with the first ANC visit preferably before 16 weeks of GA, otherwise at 16–24 weeks, the second ANC visit between 24–28 weeks, the third ANC visit between 28–32 weeks and the fourth ANC visit or last ANC visit between 32–40 weeks, preferably near to the EDD to ensure that the appropriate advice and care will be provided to prevent and manage problems such as multiple births (twins or more), post-maturity (which carries an increased risk of fetal death) and fetal mal-presentations. Therefore, based on the maternal enrollment framework criteria for this study, any mother with a GA of “*16 weeks-w*,” where “*w*” is any number of weeks less than 16, then the first voice message, which consists of reminders for the first ANC visit and information about advantage of this visit, will be sent. This voice message will be continued every 2 weeks until the end of the time frame for the first ANC visit which is 24 weeks of GA. Thus, a total of eight plus four voice messages will be sent as reminders for the first ANC visit and information about its advantages. Based on the time frame for the second ANC visit (24–28 weeks of GA), the 13th voice message from the initial message will be a reminder for the second ANC visit and information about its advantages. In this time frame, a maximum of two voice messages will be sent. The third stage of the voice message will be about reminders for the third ANC visit and its advantages. This stage of messaging will start from the 15th voice message and a total of two messages will be delivered. The last stage of voice messaging for ANC visit reminders and about its advantages will be started from the 17th voice message and this will consist of three parts (reminders for the fourth ANC visit, EDD and advantages of the fourth ANC and institutional delivery). A maximum number of voice messages from the date of enrollment and until the 42nd week of GA will be 21 voice messages. After the 21st voice message, the message will be changed to reminders for PNC follow-up and about its advantages. A total of three voice messages will be sent for PNC follow-up reminders and its advantages. Hence, an overall number of voice messages which will be sent for the intervention purpose will be 24. The content of all the messages will be first prepared in English and translated into the local language, “Afan Oromo,” and scripted to a female voice through a smart phone recording. Finally, these messages will be loaded on the mobile phone in voice message form to be sent regularly every 2 weeks at 7:00 a.m. to the intervention group:

“Hello, this is voice message from a health center. How are you? We are sending this message to remind you about your first ANC visit. Maternal health care services are given free of any cost. This service will improve your health and your baby’s health. Please, come for your first antenatal care visit before 4 months (16 weeks), otherwise before 6 months (24 weeks) of your pregnancy. When you came for your first ANC service you will have a medical check-up, you will receive TT vaccine (medication that prevents tetanus from you and your baby), and you will also be given iron (element) and folic acid (medication which prevents anemia during pregnancy and promotes normal fetal growth). We are waiting for you to give free maternal health care services. If you have any problem please call to 09x……..”

*The above message will be sent to the intervention group in the form of a voice message for the first ANC visit.*

“Hello, this is voice message from a health center. How are you? We are sending this message to remind you about your second ANC visit. Maternal health care services are given free of any cost. This service will improve your health and your baby’s health. Please, come for your second ANC visit before 7 months (28 weeks) of your pregnancy. When you came for your second ANC you will have a medical check-up and your baby’s conditions will be checked. You will receive another dose of TT vaccine (TT2) (medication that prevents tetanus from you and your baby) and you will also receive other medical support based your health condition. Please, remember also that your Expected Date of Delivery/EDD is DD/MM/YY. We are waiting for you to give free maternal health care services. If you have any problems pleases call to 09x……..”

*The above message will be sent to intervention group in the form of voice message for the second ANC visit.*

“Hello, this is a voice message from a health center. How are you? We are sending this message to remind you about your third ANC visit. Maternal health care services are given free from any cost. This service will improve your health and your baby’s health. Please, come for your third ANC before 8 months (32 weeks) of your pregnancy. When you came for your third ANC, you will have a medical check-up for your baby’s condition and yourself. Please, remember also that your EDD is DD/MM/YY. We are waiting for you to give free maternal health care services. If you have any problem pleases, call to 09x…….”

*The above message will be sent to the intervention group in the form of voice message for the third ANC visit*

“Hello, this is a voice message from a health center. How are you? We are sending this message to remind you about your fourth ANC visit. Maternal health care services are given free from any cost. This service will improve your health and your baby’s health. Please, come for your fourth ANC before 10 months (40 weeks) of pregnancy or near to your EDD. When you came for your fourth ANC, you will have a medical check-up for your baby’s condition and yourself. Please, remember also your EDD is DD/MM/YY. We are waiting for you to give free maternal health care services. If you have any problem please call to 09x……”

*The above message will be sent to the intervention group in the form of voice message for the fourth ANC visit*

“Hello, this is voice message from a health center. How are you? We are sending this message to remind you about your postnatal visit. Maternal health care services are given free from any cost. This service will improve your health and your newborn baby’s health. Please, come for postnatal care (PNC). When you came for PNC, you will have a medical check-up for your newborn baby’s condition and yourself. Your baby will receive vaccine (medication that prevents babies from getting different diseases) and your baby’s growth status will be checked. We are waiting to give free maternal health care services. If you or your baby have any problem please call to 09x…..”

*The above message will be sent to the intervention group in the form of a voice message for PNC visit*

#### Control arm B

Participants in the control group will receive existing SOC which informs them about the advantages of maternal health care service use at HCs, informing them about the service which is given free of charge and giving appointments for regular follow-up. Unlike the intervention group, however, these participants will not be sent the voice message to remind for appointment and information about the advantage of maternal service use. With regards to the data collections, a house-to-house survey will be made among the households which will be identified with pregnant mothers who fulfill the inclusion and enrollment criteria. The three phases of the survey (I, II and III) will be made to collect data from the mothers who are in the trial. The survey will focus on maternal socio-demographic status, maternal health conditions, nutritional status, and health care service use and pregnancy outcome. The data collection tools will be the same for both trial arms (Trial arms A and B), except for mobile-phone-based voice message status.

For either intervention or control group if the study participants move from the study area before the study is completed, or withdraw from the study before the final data collection period, they will be registered as dropouts to follow-up.

Assuming that all mothers may give birth at different GAs their status of delivery will be tracked by fieldworkers and health professionals at selected HCs (Fig. 2).

### Variables of the study

#### Primary outcome variables

Proportion of ANC service use

Proportion of institutional delivery

#### Secondary outcome variables

Proportion of PNC service use

Pregnancy outcome

#### Independent variables

Mobile-phone-based voice message

Socio-demographic characteristics (covariate)

Maternal health status (covariate)

### Operational definitions

- *Mobile-health* **=** the use of a mobile phone to support health care at individual, community or systems level
- *Maternal health care service utilization* **=** the women’s health care service use from conception to delivery and during postnatal period till 45 days or 6 weeks
- *Antenatal care service use* **=** maternal health care service use during pregnancy
- *Institutional delivery* **=** giving birth in health facilities
- *Postnatal care service use* = maternal health service use after birth within 45 days
- *Pregnancy outcome* ***=*** the final result of a fertilization event such as live birth (full-term or preterm birth), stillbirth, spontaneous abortion and induced abortion
- *Adverse pregnancy outcome* = includes maternal adverse outcomes and/or neonatal adverse outcomes
- *Maternal adverse outcome* = maternal illness or maternal near miss and/or maternal death
- *Neonatal adverse outcome* = neonatal morbidity and/or neonatal mortality
- *Stillbirth* **=** any fetus born at 28 weeks of gestational age or more with no heart beat or respiratory effort
- *Low birth weight* **=** birth weight less than 2500 g after viability
- *Preterm* **=** any birth occurring between 28 and 37 weeks of gestation
- *Abortion* **=** the termination of pregnancy before 28 weeks of GA
- *Maternal near miss* **=** a woman who nearly died but survived a complication that occurred during pregnancy, childbirth or within 45 days of termination of pregnancy
- *Maternal mortality* **=** the death of women during pregnancy or within 45 days of termination of pregnancy irrespective of the duration from any cause related to, or aggravated by, the pregnancy or its management but not from accidental or incidental causes
- *Neonatal mortality* **=** the death of infants with in the first 28 days of life
- *Institutional delivery* = child-birth attended by skilled health professionals
- *Maternal under-nutrition* **=** mother will be considered as undernourished if she has anemia and/or wasting or/and underweight or low weight-gain during pregnancy
- *Anemia* **=** mother will be considered as anemic if the level of hemoglobin is less than 11 g/dl which will be measured through the Hemocue machine
- *Severe anemia* = mother will be considered to have severe anemia if the level of hemoglobin is less than 7 g/dl which will be measured through Hemocue machine
- *Wasting* = mother will be considered to have wasting if the MUAC measurement is less than 18.5 cm
- *Underweight* = mother will be considered as underweight if her Body Mass Index (BMI) is less than 18.5 kg/m^2^

#### Outcome measures

The baseline characteristics of the study participants in both intervention and control groups will be compared with those of the general population. During baseline assessment, socio-demographic characteristics, eligibility criteria (pregnancy with less than 16 weeks of GA, ownership of a mobile phone and willingness to participate) and maternal health conditions, Nutritional status and previous maternal health care use experiences will be assessed.

#### Primary outcome measures

This study has two primary outcomes (proportion of ANC use and institutional delivery). The assessment of outcome measures will be conducted starting from phase II data collection time (after the eighth voice message or 4 months from the date of enrollment) and at phase III data collection time (after the 24th voice message or at close-out time off study). All the participants in the trials will be interviewed about their ANC visits and place of delivery by using a structured questionnaire. Antenatal care service use is defined as maternal health care service use during pregnancy. The participants will be considered as an ANC service user if they have at least one ANC visit. Institutional delivery is giving birth at a health facility (Fig. 3).

#### Secondary outcomes

The secondary outcome measures consist of PNC service use and pregnancy outcomes. Postnatal care service use is a maternal health care service use after birth within 45 days or 6 weeks. The participants will be considered as a user of PNC services if they have at least one PNC follow-up. Pregnancy outcome is the final result of fertilization events such as a live birth (full term or preterm), still birth, spontaneous abortion or induced abortion, low birth weight and normal birth weight. All the participants of this trial will be interviewed for the secondary outcome at phases II and III data collection time. To assess secondary outcome measures, a structured, interview-based questionnaire, which includes a status of PNC follow-up and pregnancy outcome, will be used.

For both primary and secondary outcome measures, participants’ medical record will be used for cross checking to avoid social desirability effects during the interview (Fig. 3).

### Analysis plan: analysis of primary and secondary outcome

In this study, data will be collected at three phases. Phase I data collection will be done during enrollment time; phase II data collection will be conducted after 4 months of enrollment or after the eighth voice message; and end-line data collection or close-out time data collection (phase III data collection time) will be made after the 24th voice message. All the data will be checked for completeness at the time of data collection, coded and entered into Epi-Info-7 by using double-data entry method and they will be transported to STATA software Version 11 for analysis. Data cleaning will be done by the running of simple frequency distributions for internal consistency.

The report of this findings will be made according to the Consolidated Standards of Reporting Trials (CONSORT) standards for reporting RCTs. This is a behavioral intervention which is unlikely to cause adverse effects. Hence, the analysis will be done after the close-out time of the data collection. Intention-to-treat (ITT) principles will be used for primary outcome analysis; therefore, all the participants will be analyzed according to the arms they will be randomized to. During ITT analysis, the participants lost to follow-up, resulting in missing to maternal health care service use at close-out time of data collection, will be considered as non-health care service users.

#### Sensitivity and per-protocol analysis

Sensitivity analysis will be conducted by including only participants who completed the 24th voice message follow-up. A per-protocol analysis will be done to assess the effect of the intervention among the participants who have full follow-up with maternal health care service use. The participants who come for all ANC visits (first to fourth ANC visits) within the recommended time frame for ANC visits, and give birth at a health facility, will be considered as good protocol adherents for the primary outcome. The participants who do not come for ANC visits on the recommend time frame, mainly after the recommended time frame for ANC visits, and/or give birth out of a health facility, will be considered as poor adherents. Those participants who never come for ANC visits and give birth out of a health facility will be considered as non-adherents and not included in the sensitivity analysis.

#### Sub-group analysis

Exploratory sub-group analysis will be done to identify whether the effects of intervention vary with socio-demographic characteristics, maternal health condition, nutritional status and previous health care service use difference among the participants. If statistically significant heterogeneity is identified, then relative risk with 99% confidence intervals will be estimated.

### Statistical method

For primary and secondary outcomes, Relative Risk with 95% confidence interval will be estimated. The association of dependent and independent variables will be checked by using the chi-square test. For count data, further analysis will be done by using multivariate count data logistic regressions or the Poisson Regression Model will be used to examine the effect of treatment on trial arms and Kaplan-Meier survival analysis will be used to compare maternal health care service use over time among treatment and control groups. Moreover, to adjust for both the clustering effect of the districts with their respective HCs and the participant-level intracluster correction, Generalized Estimating Equation (GEE) models will be used. All the analysis will be done by using STATA software.

### Data quality control

In this investigation, mobile-phone-based voice message will be used for reminders of ANC and PNC follow-up appointments and EDD with information about their advantages. This message will be sent in the local language (Oromiffa) and integrated into regular automatic mobile voice messaging at every 2-week interval. The content of the voice messages will be recorded on m-health mobile phones for all who are conducting the study for pregnant mothers under the interventions. Moreover, all enrolled mothers will be oriented as they will receive voice messages regularly from the date of enrollment for appointment reminders.

Data collectors will be given a 5-day intensive training on the contents of data collection tools and data collection procedures. The questionnaire will be prepared in English and translated into the local language for interview purposes and back into the English language for data analysis and to maintain consistency of information. The data will be checked for completeness at all stages of the data collection and possible errors will be returned to the data collectors for correction. Furthermore, a focal person will be assigned to study sites throughout the study period as supervisors and, every 2 weeks, the activities will be audited by the investigators and recorded. Network and electricity challenges will be settled with a systematic way of shifting time of communication without deleting the number of communications. The pre-test will be done on 5% of the total sample size away from the actual study site. Moreover, an independent quality control team will monitor the quality of the treatment or intervention. This team will visit the study process three times (at the start of the intervention, midterm of the intervention and at the end line) until the investigation is completed.

## Discussion

Mobile technology has greatly improved health care service provision at the individual, community and system level. Related with this, few studies focusing on adherence to maternal health care service use have been done in sub-Saharan African (SSA). In previous studies, participants have been selected from health facilities [16, 25–31], whereas the current study will focus on the intervention of mobile-phone-based voice messages for early initiation of maternal health care service use and its uptake. In this investigation, the study subjects will be recruited from the community by using a pregnancy screening test (HCG urine test) and portable U/S to include only those mothers with a GA of less than 16 weeks. This GA is preferred because it is recommended that all pregnant mothers should have their first ANC visit within first 12 weeks of GA, otherwise before 24 weeks.

More specifically, this investigation may uncover concerns regarding poor maternal health care service utilization unique to low-resource settings and will influence m-health program implementation, assuming that this trial demonstrates effectiveness.

There is also limited evidence which shows the effectiveness of m-health on maternal health care service use in low- and middle-income countries. Thus, mobile-phone-based voice message trial study is carefully designed with the assumption of achieving high maternal care service utilization among treatment group as compared to control. In turn, the intervention should reduce adverse pregnancy outcomes.

Randomized controlled trial studies, which were conducted in Zanzibar, Kenya and Nigeria at different times to evaluate the effect of m-health on maternal health care service utilization, have shown a promising outcome on maternal health care service utilization [16, 28, 32, 33]. However, in all aforementioned investigations the study participants were recruited from health facilities which may not show the effect of m-health on early initiation of maternal health care service use. Thus, for the current study, recruitment and enrollment of the study participants will be conducted at the community level. In addition, this investigation will enroll only those mothers at less than 16 weeks of GA to test the effectiveness of m-health on early initiation of maternal health care service use and its uptake. It is also recognized that m-health is a dynamic issue with the fast transfer in both technology and information literacy for developing countries. Hence, there is a need to examine what works or does not work yet and to establish a standard in this area. Therefore, this mobile-phone-based voice message trial study should generate strong evidence on maternal health care service utilization in resource-limited settings with poor literacy, which can be generalizable to similar populations in various settings.

### Trial status

Implementation of the study will be started in August 2017. At this time participant selection has not started.

## Additional file

Additional file 1: SPIRIT 2013 Checklist: recommended items to address in clinical trial protocol. (DOC 251 kb)

## Abbreviations

ANC: Antenatal care
BEmONC: Basic Emergency Obstetrics and Newborn Care
CONSORT: Consolidated Standard of Reporting for Trials
EDD: Expected date of delivery
EDHS: Ethiopian Demographics Health Survey
GEE: Generalized Estimating Equation
HC: Health centers
HCG: Human chorionic gonadotropin
HEP: Health Extension Program
HEWs: Health extension workers
HIV: Human immunodeficiency virus
HPs: Health post satellites
ICC: Intracluster correlation coefficient
ID No: Identification number
IHRERC: Institutional Health Research Ethics Review Committee
ITU: International Telecommunications Union
LMP: Last menstrual period
m-health: Mobile-health
MLIC: Middle- and low-income countries
MMR: Maternal mortality rate
NMR: Neonatal mortality rate
PNC: Postnatal care
SDG: Sustainable Development Goal
SOC: Standard of care
SRS: Simple random sampling
U/S: Ultrasound
UNICEF: United Nation International Children Emergency Fund
WHO: World Health Organization
