# Supplementary material for: Effect of Mobile-health on maternal health care service utilization in Eastern Ethiopia: study protocol for a randomized controlled trial
Source: Trials. 2018 Feb 12;19:102. doi: 10.1186/s13063-018-2446-5 (PMC5809837; doi:10.1186/s13063-018-2446-5)
Supplement: Supplementary file 1 — SPIRIT 2013 Checklist: recommended items to address in clinical trial protocol. (DOC 251 kb) [file 13063_2018_2446_MOESM1_ESM.doc]

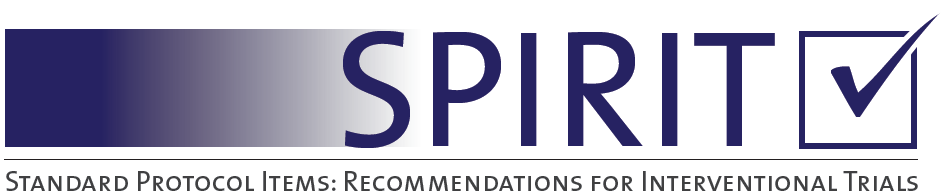


SPIRIT 2013 Checklist: Recommended items to address in clinical trial protocol

| Section/item | | | Item. No | Description | | | | |
| --- | --- | --- | --- | --- | --- | --- | --- | --- |
| **Administrative information** | | | | | | | | |
| **Title** | | | 1 | - ***Descriptive title identifying the study design, population, interventions, and, if applicable, trial acronym***   Effect of mobile-health on maternal health care services utilization in Eastern Ethiopia, Oromia region: study protocol for randomized controlled trial. | | | | |
| **Trial registration** | | | 2a | - ***Trial identifier and registry name. If not yet registered, name of intended registry***   **Identifier number:** PACTR201704002216259.This trial study was registered at Pan African clinical trial registry, [www.panctr.org](http://www.panctr.org/). | | | | |
|  | | | 2b | - ***All items from the World Health Organization Trial Registration Data Set***   All items from the World Health Organization Trial Registration Data Set which are applicable for this study are included. | | | | |
| **Protocol version** | | | 3 | - ***Date and version identifier***   **Date registered:** This trial study was registered on 2017/04/19 as primary trial study with following public and Official scientific titles;  **Public Title :** “M-Health and Maternal Health care Services Use”  **Official scientific Title:** “Effects of Mobile-health on maternal health care services utilizations in Haramaya and Kombolcha Districts, Oromia region, Eastern Ethiopia.” | | | | |
| **Funding** | | | 4 | - ***Sources and types of financial, material, and other support***   This study is funded by Haramaya University under Innovative research grant funding opportunity and the funding organization give us only financial support. The funding identification code or funding reference number which was given by funding organization is: ***HURG-2016-03-04-04.*** | | | | |
| **Roles and responsibilities** | | | 5a | - ***Names, affiliations, and roles of protocol contributors***   **1. Tilayie Feto Gelano (MSc. BSc)**: Haramaya University, College of Health and Medical Sciences, Department of Paediatrics and Neonatal Nursing, School of Nursing and Midwifery, Harar Ethiopia, he is the principal investigator of this study, wrote initial draft of the proposal, developed the protocol and will supervise the implementation of study.  **2. Nega Assefa (Ph.D., MPH. BSc):** Haramaya University, College of Health and Medical Sciences, He is co-investigator of this study and participated in designing study protocol, and responsible for the statistical design of the trial and data analysis.  **3. Yadeta Dassie Bacha (Ph.D., MPH, BSc):** Haramaya University, College of Health and Medical Sciences, He is co-investigator of this study and participated in designing study protocol, and responsible for the statistical design of the trial and data analysis.  **4. Afendi Abdi Mahamed (MSc. BSc):** Haramaya University, College of Sciences and Technology, Department of Computer and Information Sciences. He is co-investigator of this study and participated in designing study protocol, and responsible for the statistical design of the trial, data analysis and installing of Open Data Kit (ODK) for data collection, and Installing /loading of voice message on mobile phone in collaboration with local telecommunication.  **5. Kedir Teji Roba (Ph.D., MPH., BSc):** Haramaya University, College of Health and Medical Sciences, He is co-investigator of this study and participated in designing study protocol, and responsible for the statistical design of the trial and data analysis.  **6. Mitiku Teshome Hambisa (MPH. BSc):** Haramaya University, College of Health and Medical Sciences, He is co-investigator of this study and participated in designing study protocol, and responsible for the statistical design of the trial and data analysis. | | | | |
|  | | | 5b | - ***Name and contact information for the trial sponsor***   Funding Organization for this study is Haramaya University, Research Office, Eastern Ethiopia, Phone: +251-025-5530332 , P.O.Box 138 , Dire Dawa, Ethiopia. Currently, there is no other Organization which Sponsor this study. | | | | |
|  | | | 5c | - ***Role of study sponsor and funders, if any, in study design; collection, management, analysis, and interpretation of data; writing of the report; and the decision to submit the report for publication, including whether they will have ultimate authority over any of these activities***   The funding organization has no role in designing of study protocol, writing and its submission for publication, analysis of data, its interpretation and decision to submit the report for publication.  Funding organization (Haramaya University) will have a role in monitoring of data collection and intervention process as per the University’s granted research monitoring principle. | | | | |
|  | | | 5d | - ***Composition, roles, and responsibilities of the coordinating centre, steering committee, endpoint adjudication committee, data management team, and other individuals or groups overseeing the trial, if applicable (see Item 21a for data monitoring committee)***   As it was mentioned, most of the activities such as supervising of data collection, Monitoring of interventions, and data processing and analysis and reporting of the results will be performed by investigators.  In addition, it is planed that three independent quality controlling people will monitor the quality of the treatment or interventions and data collections. This team will be selected based on the following qualification and composition.  1. One person who has at least Master degree (MSc) in Maternal and Reproductive Health with at least Assistant professor rank.  2. One person who has at least Master degree in Epidemiology/in Health research or related field of study with at least Assistant professor rank.  3. One person from Ethics review team Or Institutional Health Research Ethics Review Committee (IHRERC).This team will visit study process three times (at starting of intervention, midterm of intervention and at end line) until the investigation will be completed.  On top of this, funding organization (Haramaya University) will take role in monitoring of data collection and intervention process, this is outside of the study and they visit the data collection process and intervention based on the University’s schedule for monitoring and supervising of granted research. | | | | |
| Introduction | | |  |  | | | | |
| **Background and rationale** | | | 6a | - ***Description of research question and justification for undertaking the trial, including summary of relevant studies (published and unpublished) examining benefits and harms for each intervention***   Mobile phone, which has reached the hands of 90% of the world’s population, of whom 80% are rural dwellers [1], has also made possible Mobile-health/M-health, the usage of mobile technology to improve health. Globally, the fast development of mobile technology has generated new ways to address public health challenges and shifted the paradigm of health care access and delivery[1, 2]. In the health care system of developing countries, the mobile phone technology is used to report health information, health care is delivered through telemedicine to population otherwise deprived, improve adherence to treatment and appointments [3]. M-health also plays a big role in sexual and reproductive health care and seen as a key area in achieving the global strategy for women’s and children’s health[4]. Evidence has shown that m-health minimizes time barrier and facilitates urgent care during emergency obstetric referrals and maternal health care use in LMIC[5].  Developing regions accounted for approximately 99% of global maternal deaths in 2015, of which Sub-Saharan Africa alone accounted for about two thirds, followed by one third in Southern Asia. Achieving the Sustainable Development Goals (SDG) target of a maternal mortality rate (MMR) below 70 per 100,000 live births will require 7.5% reduction of MMR every year between 2016 and 2030 [6]. This will need more than three times the annual rate of global maternal mortality reduction which was observed between 1990 and 2015 [7]**.** Furthermore, three million babies die every year before they are a month old, and a similar number are stillborn [8, 9]**.** Most of the deaths are due to lack of quality maternal and neonatal health care service provision [10]**.**  At the moment, many countries, including Ethiopia, are working to meet the recommendation of having skilled birth attendants for all births [11]**.** However, maternal health problem is still very serious; every year about 350,000 maternal deaths occur because of pregnancy and its related complications. The problem is more serious in developing countries[11, 12]. In Ethiopia, the mortality rate increased from 673 per 100,000 live births in 2005 to 676 per 100,000 live births in 2011[13].  Since 2003, Ethiopia has been increasing access to primary health care service through Community Based Health Extension Program (CBHEP). Between 2003 and 2010, about 34,000 health extension workers (HEWs) were trained and deployed in 15,000 kebeles (villages), each of which had one newly constructed health post(HP) and about 3,000-5,000 dwellers [12]. Regardless of all these efforts, according to Ethiopian Demographics Health Survey (EDHS) 2011, the number of women who had antenatal care (34%), institutional delivery (10%), and postnatal care (6%) service by skilled workers were very small, and this has resulted in high maternal and neonatal morbidity and mortality[11].To tackle this problem more effectively, practicing innovative way of health care service delivery which will be integrated with the existing health care system is crucial, and thus mobile technology is being used. Despite the widespread use of mobile technologies in health care system, there is limited evidence on the efficacy of mobile phone based intervention or m-health on maternal health care service utilization. In Ethiopia, no study has been conducted on the topic. To fill the gap, this study, through randomized controlled trial (RCT), examines the effects of m-health on maternal health care service use in Eastern Ethiopia. It is hypothesized that m-health will increase maternal health care service provision in limited resource and literacy. In this RCT, two districts with 34 kilometres apart will be selected to reduce the possible risk of contamination between intervention and control arms and to evaluate the barriers of implementation. The outcomes of this investigation will be analysed for both intervention and control arms separately. Examining challenges and outcomes at this stage will provide insight about m-health implementation for improved maternal health care services use. | | | | |
|  | | | 6b | - ***Explanation for choice of comparators***   This study will be conducted in Eastern Hararghe zone, Oromia Regional state, Eastern Ethiopia.  Eastern Hararghe zone has fifteen districts among which, Haramaya and Kombolcha districts are selected by the purposive method as primary study unit. The main reason for this purposive selection of the two districts is accessibility of electric power and network which will be mandatory for mobile phone charging and communication during the study period. In addition, the community in these two districts has similar socio-demographic characteristics and maternal health care service coverage [14, 15]. Then, by using random allocation (Lottery method) the two districts are assigned to intervention and control group. Accordingly, Haramaya district is allocated to an intervention group (m-health+ Standard of Care) whereas Kombolcha district is assigned to control group (Standard of Care). The comparators or control group /pregnant women from Kombolcha district will receive only the existing standard of care such as counseling of mothers about the advantage of maternal health care services use at health centers, informing them about service which is given free of charge and giving appointments. | | | | |
| **Objectives** | | | 7 | - **Specific objectives or hypotheses**  1. To Examine the effects of m-health on maternal Antenatal care services use 2. To Determine the effects of mobile-health on institutional delivery 3. To Examine the effects of mobile-health on maternal postnatal care services use 4. To evaluate effects of mobile -health on pregnancy outcome. | | | | |
| **Trial design** | | | 8 | - ***Description of trial design including type of trial (e.g., parallel group, crossover, factorial, single group), allocation ratio, and framework (e.g. superiority, equivalence, no inferiority, exploratory)***   Single-blind, cluster randomized, controlled trial study will be conducted by using districts and respective health centres as the unit of randomization. Participants will be randomized (allocated by lottery method) to mobile-phone based intervention (voice message for reminding and information about advantage of maternal health care services use) or Standard of care /control (no voice message for reminding and information about advantage of services use) under their respective districts and health centres with 1:1 allocation ratio (Figure 1). | | | | |
| Methods: Participants, interventions, and outcomes | | | | | | | | |
| **Study setting** | | | 9 | - ***Description of study settings (eg, community clinic, academic hospital) and list of countries where data will be collected. Reference to where list of study sites can be obtained***   This study will be done in Haramaya and Kombolcha Districts, which are in Eastern Haaraghe Zone, Oromia Regional State, Eastern Ethiopia.  Haramaya District, which is about 506 km from Addis Ababa, the capital city of Ethiopia, is located between [9°24′N 42°01′E](https://tools.wmflabs.org/geohack/geohack.php?pagename=Alemaya&params=9_24_N_42_01_E_region:ET-OR_type:city(15317)) and 1400 -2340 meters above sea level. According to the 2007 national census of the country, its population was 271,018, of whom 48.97% were females and 18.46% were urban dwellers. Moreover, by using pregnancy estimation factors of 4.6 %, around 12,467 women are expected to be pregnant in this district. In the district, there are seven health centres, twenty two health posts and one hospital, [16]**.**  On the other hand, the district of Kombolcha is 540 km from Addis Ababa and found between 1200 and 2460 meters above sea level. According to 2007 national census report, its population was about 140,080, of whom 49.3% were females and 9.01% were urban dwellers. Moreover, by using pregnancy estimation factors of 4.6%, about 6,444 women are expected to be pregnant in this district. In Kombolcha, there are five health centers and twenty two health posts.  Related with study parameters like socio-demographic, Economic characteristics and Maternal health care services coverage the two districts have similarity [17]. | | | | |
| **Eligibility criteria** | | | 10 | - ***Inclusion and exclusion criteria***   **Participants: Inclusion and exclusion criteria**  The Participants will be recruited from two districts (Haramaya and Kombolcha Districts) based on pre-set eligibility criteria (pregnancy, gestational age, ownership of mobile phone and willingness to participate). The participant will be included in the trial if gestational age is less than sixteen weeks, own mobile phone and has willingness to participate. The Potential trial participants will be identified by the flied workers based enrolment criteria. For all eligible participants, the flied workers will explain about study by reading the participants information sheets; if the study subjects are willing to participate, written informed voluntary consent will be obtained and baseline data collection will be made.  In this trial study women who fulfil enrolment criteria for the study but critically ill at time of enrolment will be excluded from the study. Critically ill women means those pregnant mothers who unable to respond to interview questions because of illness. In addition, women with any systemic illness during this time will be excluded from study. Pregnant women with any systemic illness need a special ANC follow up which will set precondition for the women to have good maternal health care services use and hence excluded to reduce the effects of pre-set condition. | | | | |
| **Interventions** | | | 11a | - ***Interventions for each group with sufficient detail to allow replication, including how and when they will be administered***   **Intervention and follow up procedures**  This study will have two trial arms, “Trial Arm A” is the intervention and “Trial Arm B” is the control group.  **Trial Arm A:** In this arm, mobile phone based voice message consisting of information about the advantage of maternal health care service use (advantage of ANC, PNC and institutional delivery) and reminders for appointment will be sent. Particularly, every two weeks a voice message composed of reminders for ANC follow up and information about its advantages (first to fourth ANC visits) and EDD will be sent. After birth, reminders for PNC follow up (first to third PNC follow up) and information about its advantages will be sent as voices message through the mobile phone.   - Published evidence supports that the first ANC visit should be as early as possible in pregnancy, preferably in the first trimester (within the first twelve weeks of gestation) and in a normal pregnancy the protocol for pregnancy care recommends four ANC visits [18]. Hence, appointments for ANC follow-up will be adjusted based on gestational age with the first ANC visit preferably before 16 weeks of gestational age, otherwise at 16-24 weeks, the second ANC visit between 24-28 weeks, the third ANC visit between 28-32 weeks and fourth ANC visit or last ANC visit between 32-40 weeks, preferably near to the EDD to ensure that the appropriate advice and care will be provided to prevent and manage problems such as multiple births (twins or more), post maturity (which carries an increased risk of fetal death) and fetal mal-presentations. Therefore, based on the maternal enrolment framework criteria for this study, any mother with gestational age of “***16 weeks-w”***, if ***“w”*** will be any number of weeks less than 16, then the first voice message which consists of reminders for the first ANC visit and information about advantage of this visit will be sent. This voice message will be continued every two weeks until the end of time frame for the first ANC visit which is 24 weeks of GA. Thus, a total of eight plus four voice messages will be sent as reminders for the first ANC visit and information about its advantages. Based on the time frame for the second ANC visit (24-28 weeks of GA), the thirteenth voice message from the initial message will be a reminder for the second ANC visit and information about its advantages. In this time frame, a maximum of two voice messages will be sent. The third stage of voice message will be about reminders for the third ANC visit and its advantages. This stage of messaging will start from the fifteenth voice message and a total of two messages will be delivered. The last stage of a voice messaging for ANC visit reminders and about its advantages will be started from the seventeenth voice message and this will consist of three parts (reminders for the fourth ANC visit, EDD and advantages of the fourth ANC and institutional delivery). A maximum number of voice messages from date of enrolment and until the 42^nd^ week of GA will be 21 voices messages. After the 21^st^ voice message, the message will be changed to reminders for PNC follow up and about its advantages. A total of three voice messages will be sent for PNC follow-up reminders and its advantages. Hence, an overall number of voice messages which will be sent for the intervention purpose will be 24. The content of all the messages will be first prepared in English and translated into the local language “Afan Oromo” language and scripted to a female voice through smart phone recording. Finally, these messages will be loaded on the mobile phone in voice message form to be sent regularly every two weeks at 7:00 AM to the intervention group. - The following texts are the contents of messages which will be translated into local language, Recorded by Smart Phone or scripted Female Voice and loaded on mobile phone as voice message and sent to intervention group every two weeks.     *“Hello, this is voice message from a health center. How are you? We are sending this message to remind you about your 1^st^ ANC visit. Maternal health care services are given free of any cost. This service will improve your health and your baby’s health. Please, come for your first Antenatal care visit before 4 months (16 weeks), otherwise before six months (24 weeks) of your pregnancy. When you came for your 1^st^ ANC service you will have a medical check-up, you will receive TT vaccine (medication that prevents tetanus from you and your baby), and you will also be given iron (element) and folic acid (medication which prevents anaemia during pregnancy and promotes normal fetal growth). We are waiting for you to give free maternal health care services. If you have any problem please call to 09x……..”*  ***The above message will be sent to the intervention group in the form of a voice message for the 1^st^ ANC visit.***  *“Hello, this is voice message from a health center. How are you? We are sending this message to remind you about your second ANC visit. Maternal health care services are given free of any cost. This service will improve your health and your baby’s health. Please, come for your second ANC visit before 7 months (28 weeks) of your pregnancy. When you came for your second ANC you will have a medical check-up and your baby’s conditions will be checked. You will receive another dose of TT vaccine (TT2) (medication that prevents tetanus from you and your baby) and you will also receive other medical support based your health condition. Please, remember also that your Expected Date of Delivery/EDD is DD/MM/YY. We are waiting for you to give free maternal health care services. If you have any problems pleases call to 09x……..”*  ***The above message will be sent to intervention group in the form of voice message for 2^nd^ ANC visit.***  *“Hello, this is a voice message from a health center. How are you? We are sending this message to remind you about your third ANC visit. Maternal health care services are given free from any cost. This service will improve your health and your baby’s health. Please, come for your third ANC before 8 months (32 weeks) of your pregnancy. When you came for your third ANC, you will have a medical check-up for your baby’s condition and yourself. Please, remember also that your EDD is DD/MM/YY. We are waiting for you to give free maternal health care services. If you have any problem pleases, call to 09x…….”*  ***The above message will be sent to the intervention group in the form of voice message for the 3^rd^ ANC visit***  *“Hello, this is a voice message from a health center. How are you? We are sending this message to remind you about your fourth ANC visit. Maternal health care services are given free from any cost. This service will improve your health and your baby’s health. Please, come for your fourth ANC before 10 months (40 weeks) of pregnancy or near to your EDD. When you came for your fourth ANC, you will have a medical check-up for your baby’s condition and yourself. Please, remember also your EDD is DD/MM/YY. We are waiting for you to give free maternal health care services. If you have any problem please call to 09X……”*  ***The above message will be sent to the intervention group in the form of voice message for 4^th^ ANC visit***  *“Hello, this is voice message from a health center. How are you? We are sending this message to remind you about your postnatal visit. Maternal health care services are given free from any cost. This service will improve your health and your Newborn baby’s health. Please, come for Postnatal care (PNC). When you came for PNC, you will have a medical check-up for your Newborn baby’s condition and yourself. Your baby will receive vaccine (medication that prevents babies from getting different diseases) and your baby’s growth status will be checked. We are waiting to give free maternal health care services. If you or your baby have any problem please call to 09X…..”*  ***The above message will be sent to the intervention group in the form of a voice message for PNC visit***  ***Control Arm B:*** Participants in the control group will receive existing standard of care (SOC). Which inform about the advantages of maternal health care service use at health centers, informing them about service which is given free of charge and giving appointments for regular follow up. Unlike the intervention group, however, these participants will not be sent the voice message to remind for appointment and information about the advantage of maternal services use. With regards to the data collections, a house to house Survey will be made among the households which will be identified with pregnant mothers who fulfil the inclusion and enrolment criteria. The three phases of survey (I, II and III) will be made to collect data from the mothers who are in the trial. The survey will focus on maternal socio-demographic status, maternal health conditions, nutritional status, and health care service use and pregnancy outcome. The data collection tools will be the same for both trial arms (arms A and B), except for mobile phone based voice messages status.  For either intervention or control group if the study participants move from the study area before the study is completed, or withdraw from the study before the final data collection period, they will be registered as dropouts to follow up.  Assuming that all mothers may give birth at different gestational ages their status of delivery will be tracked by fieldworkers and health professionals at selected health centers. | | | | |
|  | | | 11b | - ***Criteria for discontinuing or modifying allocated interventions for a given trial participant (e.g., drug dose change in response to harms, participant request, or improving/worsening disease)***   In this study, the interventions has minimal risk or no side effect on study subject, Hence, the intervention will be discontinued only if the study participants move from the study area before the study will be completed, or withdraw from the study before final data collection time. Otherwise, there is no other criteria to modify or discontinue the interventions. For either intervention or control groups if the study participants move from the study area before the study will be completed, or withdraw from the study before final data collection time, they will be registered as a dropout to follow up. | | | | |
|  | | 11c | | | - ***Strategies to improve adherence to intervention protocols, and any procedures for monitoring adherence (e.g., drug tablet return, laboratory tests)***   The concern of adherence to intervention protocols in this study might be related with Network and electricity challenges which will be settled with a systematic way of shifting time of communication without deleting a number of communications. Moreover, the time of voice message delivery will be adjusted to the same time for all mothers under intervention group. Specifically, it will be send at 7:00 AM in the morning to increase access to the voice message.  With regards to data collections, house to house survey will be conducted among those households which identified with pregnant mothers who will fulfil inclusions and enrolment criteria. Threes phase of survey (phase I, II and III) will be conducted to collect data from study participants. This survey will focus on maternal Socio-Demographic status, maternal health condition, nutritional status, and health care service use status, maternal awareness about maternal health care service use, maternal awareness about pregnancy related dangers signs and pregnancy outcome. Data collection tools will be the same for both trial arms (arm A and B) except for mobile phone based voice messages. | |  | |
|  | 11d | | | | | - ***Relevant concomitant care and interventions that are permitted or prohibited during the trial***   Relevant concomitant care and interventions that will be permitted for both group is standard of care.   - **Prohibited treatment during the trial period:**   Any mobile phone based advices for care, reminding of appointment of follow-up for maternal health care and other services which will be given through mobile phone is not allowed to a control group. | |  |
| **Outcomes** | | | 12 | - ***Primary, secondary, and other outcomes, including the specific measurement variable (e.g., systolic blood pressure), analysis metric (e.g., change from baseline, final value, time to event), method of aggregation (e.g., median, proportion), and time point for each outcome. Explanation of the clinical relevance of chosen efficacy and harm outcomes is strongly recommended***   **Primary outcome measure**  This study has two primary outcomes (proportion of Antenatal care service use and institutional delivery). Assessment of outcome measures will be conducted starting from Phase II data collection time (after 8^th^ voice message or 4 months from the date of enrolment) and at phase III data collection time (after 24^th^ voice message or at close out time off study). All participants in the trial will be interviewed by using structured questionnaire about their status of ANC visits, and place of delivery. ANC service use is defined as maternal health care service use during pregnancy. The women will be considered as ANC service user if she has at least one ANC visit. Institutional delivery is giving birth at health facilities. Finally, based on the self-reported evidence about maternal ANC visit and institutional delivery will be confirmed from card or maternal health care services use record at health facility (health center).  **Secondary outcome**  Secondary outcome measure consists of proportion postnatal care (PNC) service use and pregnancy outcomes. PNC service use is a maternal health care service use after birth within 45 days. Participants will be considered as a user of PNC services if they have at least one PNC follow up. Pregnancy outcome is the final results of fertilization events like; live birth (full term or preterm), low birth weight still birth, spontaneous or induced abortion. All participants of this trial will be interviewed for the secondary outcome at phase II and III data collection time. To assess secondary outcome measures, interview based structured questionnaire which include status of PNC follow-up and pregnancy outcome will be used.  For both primary and secondary outcome measures, participants’ medical record will be used for cross checking to avoid social desirability bias during the interview. | | | | |
| **Participant timeline** | | | 13 | - ***Time schedule of enrolment, interventions (including any run-ins and washouts), assessments, and visits for participants. A schematic diagram is highly recommended (see Figure 3 )***   Generally, Study will have been conducted from August 2017- October /2018 , for further about time frame or schedule for enrolment, interventions, and assessments is presented on SPIRIT Figure (Figure 3): | | | | |
| **Sample size** | | | 14 | - ***Estimated number of participants needed to achieve study objectives and how it was determined, including clinical and statistical assumptions supporting any sample size calculations***   A study which was conducted in Zanzibar showed that 60% of the intervention group used a health professional assisted delivery (institutional delivery), as compared to 47% in the control group; based on this evidence, sample size required for this study is calculated as follows [19].  P_1_=proportion of health professional assisted delivery in control group =47%  P_2_=proportion of health professional assisted delivery in intervention group =60%  Based on the above information, a sample size required for this study is calculated by using STATA/SE software with consideration of sample size for two-sample comparison of proportions [20].  **Assumption and hypothesis for sample size calculation:**  **H_o_**: P_1_ = P_2_, whereas P_1_ is the proportion in the intervention group (Population_1_) and P_2_ is the proportion in the control group (Population _2_), α is level of significance at 95% confidence interval, and β is power.  In clustered RCT the study subjects within the cluster are more likely to interact, respond in the same manner and no longer be act independently which can leads to a loss of statistical power. This intracluster dependence can be quantified by considering intracluster correction coefficient (ICC) and design effects (DE) [21]. Hence, the estimated sample size per group is inflated by considering ICC and DE of 1.3. Thus, the final sample size required for this study will be 320 for each control and treatment groups.  **Description of sample size calculation:** A sample size of 640 pregnant mothers (320 in each of the treatment and control group) will be sufficient to detect a clinically important difference of 13% between groups in the m-health intervention, using a two-sided-test of 80% power (β) and 5% of significance level (α) with 95% confidence interval**.** The 13% difference represents the difference between a 60% health professional assisted deliveries in the intervention group as compared to the control group [19]**.** | | | | |
| **Recruitment** | | | 15 | - **Strategies for achieving adequate participant enrolment to reach target sample size**   Based on EHSTP of 2016-2020, one Health Center (HC), which encompasses five Health Posts (HPs) in five Kebeles which consists 3,000-5,000 people each, can provide service for 15,000-25,000 people [22]**.** In Ethiopia, the estimated number of pregnancy in a given population is about 4.6% [23]. Hence, the total number of estimated pregnant mothers in five kebeles surrounding one health center is about 690-1150.  Among the seven health centers in Haramaya district, Addelle HC is selected by the purposive method as secondary study unit. The main reason for the purposive selection is accessibility of electric power and network, which will be mandatory for mobile charging and communication during the study period. Finally, all pregnant mothers who fulfill the enrollment criteria in the selected Kebele will be registered and 320 pregnant mothers will be selected for the intervention group by a simple random sampling method. Likewise, of the five health centers in Kombolcha district, Kombolcha health center is selected purposefully for the same reason, and from the surrounding kebeles, 320 mothers will be selected for the control group by simple random sampling method. | | | | |
| **Methods: Assignment of interventions (for controlled trials)** | | | | | | | | |
| **Allocation:** | | |  |  | | | | |
| **Sequence generation** | | | 16a | - ***Method of generating the allocation sequence (e.g., computer-generated random numbers), and list of any factors for stratification. To reduce predictability of a random sequence, details of any planned restriction (e.g., blocking) should be provided in a separate document that is unavailable to those who enroll participants or assign interventions***   **Screening and Enrolment procedure**: In this study, pregnancy, Gestational Age (GA) and ownership of mobile phone will be used as main selection and enrollment criteria. Hence, in the selected kebeles, first all the reproductive age women who had a history of amenorrhea for the last 28 days, who do not use any family planning, and who own mobile phone will be screened for pregnancy through HCG urine test. Then, the mothers who will be positive for the HCG-urine test will be given unique identification number (ID No) and screened for GA through LMP and /or portable Ultrasound (U/S). Next, those women with less than16 weeks of GA will be registered as illegible for the study. Finally, for each control and intervention group, 320 pregnant women who will fulfil enrolment criteria will be selected by simple random sampling method (Computer generated random table)[24]. They will be registered with their Unique Identification (ID No) and mobile phone number on a computer tablet. | | | | |
| **Allocation concealment mechanism** | | | 16b | - ***Mechanism of implementing the allocation sequence (e.g., central telephone; sequentially numbered, opaque, sealed envelopes), describing any steps to conceal the sequence until interventions are assigned***   To select required sample size from the list of all pregnant mothers who will fulfil the inclusion and enrolment criteria, computer generated random table will be used to select those participants from both intervention and control groups. The process of random allocation to treatment or control group is done at primary study unit, the allocation of districts to intervention or control group is done by lottery method (by random allocation). | | | | |
| **Implementation** | | | 16c | - ***Who will generate the allocation sequence, who will enrol participants, and who will assign participants to interventions***   The allocation of primary study unit (districts) to intervention or control group is performed by investigators through lottery method. Random selection of secondary study unit (health centres) from allocated district is also done by investigators. After identifying eligible study subjects, a random selection of study subjects will be followed by investigators through computer generated random table. | | | | |
| **Blinding (masking)** | | | 17a | - ***Who will be blinded after assignment to interventions (e.g., trial participants, care providers, outcome assessors, data analysts), and how***   Because of the nature of the study, the participants who will be involved in the intervention group will be informed about their participation in the study and as they will receive voice message every two weeks. However, the participants who will join a control group will not be informed about their counterpart. Moreover, data collectors and focal person who will monitor the intervention and data collection process will not be informed about who will exposed to mobile phone based intervention. | | | | |
|  | | | 17b | - **If blinded, circumstances under which unbinding is permissible, and procedure for revealing a participant’s allocated intervention during the trial** - This study is only partially blinded. | | | | |
| **Methods: Data collection, management, and analysis** | | | | | | | | |
| **Data collection methods** | | | 18a | - ***Plans for assessment and collection of outcome, baseline, and other trial data, including any related processes to promote data quality (e.g., duplicate measurements, training of assessors) and a description of study instruments (e.g., questionnaires, laboratory tests) along with their reliability and validity, if known. Reference to where data collection forms can be found, if not in the protocol***   Data will be collected in three phases (Phase I, II and III) using questionnaire loaded on four computer tablets, and by four trained BSc nurses and six Health Extension Workers (HEWs).  **Phase I:** In this phase, for both intervention and control groups, base line survey will be conducted on Socio-demographic characteristics and maternal health care service use via a structured interview based questionnaires which will be adopted from similar studies. Maternal health status will be assessed through Medical interview checklist and nutritional status will be screened by using Middle Upper Arm Circumference tape, Weight scale and Hemacue machine or Portable haemoglobin analysers to test for haemoglobin level.  **Phase II:** This stage of data collection will be conducted after the eighth voice messages or the fourth months of enrolment and the data collection will focus on maternal health condition, nutritional status and status of the voice messages which will be delivered to the intervention group, status of maternal health care services use among both intervention and control groups. During this period data related to maternal health conditions will be collected through the same tools which will be used in Phase I. In addition, data related to maternal health care services use status and voice message will be collected through structured interview based questionnaire with confirmation of voice messages from their mobile phone.  **Phase III:** This phase of data collection will be done at close out time of the study and it will be conducted after 24 voice messages or 45 days of post-delivery for every mother under intervention and control groups. It will focus on maternal health condition, nutritional status, health care services use, pregnancy out and the status of mobile phone based voice message since their enrolment time. Furthermore, mothers in the intervention group will be asked how voice messages helped them. | | | | |
|  | | | 18b | - ***Plans to promote participant retention and complete follow-up, including list of any outcome data to be collected for participants who discontinue or deviate from intervention protocols.***   In this study our assumption is to complement with zero participant’s withdraw from the study or very minimal lost to follow up. Because this study will have no side effects or have very minimal risk on study participants.  But in case if there will be unpreventable loss to follow up such as maternal death, Information such as cause of death, number of follow up she had received will be obtained from the relative or medical records and included in secondary outcome of this study. Other cause of lost to follow might be related with sudden changing of residences and in this case the required information will be obtained from mothers through their mobile phone by calling. Finally, during analysis intention to treat (ITT) method will be used to assess the effect of intervention on outcome variables. | | | | |
| **Data management** | | | 19 | - ***Plans for data entry, coding, security, and storage, including any related processes to promote data quality (e.g., double data entry; range checks for data values). Reference to where details of data management procedures can be found, if not in the protocol***   In this study, data will be collected at three phases. Phase I data collection will be done during enrolment time; phase II data collection will be conducted after four months of enrolment or after 8^th^ voice message; and end line data collection or close out time data collection (Phase III data collection time ) will be made after the 24^th^ voice message. All the data will be checked for completeness at the time of data collection, coded and entered into Epi-Info-7 by using double data entry method and they will be transported to STATA software Version-11 for analysis. Data cleaning will be done with running of simple frequency distributions for internal consistency. | | | | |
| **Statistical methods** | | | 20a | - ***Statistical methods for analysing primary and secondary outcomes. Reference to where other details of the statistical analysis plan can be found, if not in the protocol***   For primary and secondary outcomes, Relative Risk with 95% confidence interval will be estimated. The association of dependent and independent variables will be checked by using chi-square test. For count data, further analysis will be done by using multivariate count data logistic regressions or Poisson Regression model will be used to examine the effect of treatment on trial arms and Kaplan-meier survival analysis will be used to compare maternal health care service use over time among treatment and control groups. Moreover, to adjust for both the clustering effect of the districts with their respective health centers and the participants level intracluster correction, Generalized Estimating Equation (GEE) models will be used. All the analysis will be done by using STATA software. | | | | |
|  | | | 20b | - ***Methods for any additional analyses (e.g., subgroup and adjusted analyses)***   **Sub-group analysis**  Exploratory sub-group analysis will be done to identify whether the effects of intervention varies with socio-demographic characteristics, maternal health condition, nutritional status and previous health care services use difference among the participants. If statistically significant heterogeneity is identified, then relative risk with 99% confidence intervals will be estimated. | | | | |
|  | | | 20c | - ***Definition of analysis population relating to protocol non-adherence (eg, as randomised analysis), and any statistical methods to handle missing data (eg, multiple imputation)***   ***Sensitivity and per-protocol Analysis***  Sensitivity analysis will be conducted by including only participants who completed the 24^th^ voice message follow up. A per-protocol analysis will be done to assess the effect of intervention among the participants who have full follow up with maternal health care service use. The Participants who come for all ANC visits (first to fourth ANC visits) within the recommended time frame for ANC visits and give birth at a health facility will be considered as good protocol adherents for the primary outcome. The Participants who do not come for ANC visits on the recommend time frame, mainly after the recommended time frame for ANC visits and/or give birth out of health facility will be considered as poor adherents. Those participants who never come for ANC visits and give birth out of health facilities will be considered as non –adherents and not included in the sensitivity analysis. | | | | |
| **Methods: Monitoring** | | | | | | | | |
| Data monitoring | | | 21a | - ***Composition of data monitoring committee (DMC); summary of its role and reporting structure; statement of whether it is independent from the sponsor and competing interests; and reference to where further details about its charter can be found, if not in the protocol. Alternatively, an explanation of why a DMC is not needed***   In this study, most of monitoring and supervising of data collection and intervention process will be made by the investigators. | | | | |
|  | | | 21b | - ***Description of any interim analyses and stopping guidelines, including who will have access to these interim results and make the final decision to terminate the trial***   The termination of interventions will be made only if the participants will have no willingness to participate in the study or declare to stop the participation. In this case the termination will be made by investigators. | | | | |
| Harms | | | 22 | - ***Plans for collecting, assessing, reporting, and managing solicited and spontaneously reported adverse events and other unintended effects of trial interventions or trial conduct***   Risk related with this study is very minimal; study subject who will participate in this study will only lose few minute needed for interviews and listening to voice message send to them. Hence, there will be no harm or any side effects that may be caused due to interventions. | | | | |
| Auditing | | | 23 | - ***Frequency and procedures for auditing trial conduct, if any, and whether the process will be independent from investigators and the sponsor***   Focal person will be assigned to study sites throughout the study period as supervisors and every two weeks the activities will be audited by investigators and recorded. | | | | |
| Ethics and dissemination | | | | | | | | |
| Research ethics approval | | | 24 | - ***Plans for seeking research ethics committee/institutional review board (REC/IRB) approval***   Ethical approval for the study was granted from Haramaya University, College of Health and Medical Sciences, Institutional Health Research Ethics Review Committee (IHRERC), (Ref.No.IHRERC/127/2017), May 08/2017. | | | | |
| Protocol amendments | | | 25 | - ***Plans for communicating important protocol modifications (e.g., changes to eligibility criteria, outcomes, analyses) to relevant parties (e.g., investigators, REC/IRBs, trial participants, trial registries, journals, regulators)***   Any amendments made to this study protocol will be communicated with all concerned bodies accordingly. | | | | |
| Consent or assent | | | 26a | - ***Who will obtain informed consent or assent from potential trial participants or authorised surrogates, and how (see Item 32)***   Written informed voluntary consents will be obtained from all participants by investigators. | | | | |
|  | | | 26b | - ***Additional consent provisions for collection and use of participant data and biological specimens in ancillary studies, if applicable***   In addition, formal letter will be submitted to all concerned bodies such as districts health bureau, health centres and sub-district administrators in the study area to get their co-operation in facilitating the study. | | | | |
| Confidentiality | | | 27 | - ***How personal information about potential and enrolled participants will be collected, shared, and maintained in order to protect confidentiality before, during, and after the trial***   Any information that study participants provide us will be confidential. There will be no information that will identify individual. The finding of this study will be general for the study population and will not reflect any thing particular of individual to person. The questionnaire will be coded to exclude showing names; no references will be made in oral or written reports that could link participants to the research. Participation in this study is voluntary. The study participants have the right to declare not to participate in this study. They have the right to with draw from the study at any time without depriving any benefit they deserve. | | | | |
| Declaration of interests | | | 28 | - ***Financial and other competing interests for principal investigators for the overall trial and each study site***   All investigators declare that they have no competing of interest for overall process of this study and at each study site. | | | | |
| Access to data | | | 29 | - ***Statement of who will have access to the final trial dataset, and disclosure of contractual agreements that limit such access for investigators***   There is no any contractual agreements that limit investigators from access to the final trial dataset. Full mandate is given to the investigators for writing report and submit to any concerned body or publish the finding. | | | | |
| Ancillary and post-trial care | | | 30 | - ***Provisions, if any, for ancillary and post-trial care, and for compensation to those who suffer harm from trial participation***   As it was mentioned, this study has very minimal harm to the study participants,  There is no plan for provision of compensation to the study participants, rather if the finding of this study will have positive effects on maternal health care services use the intervention will be initiated for all population those who are located in the study area. Moreover, if woman is identified with any health problems during this study period, she will be referred to other health facilities for better care with transportation arrangement by investigators. | | | | |
| Dissemination policy | | | 31a | - ***Plans for investigators and sponsor to communicate trial results to participants, healthcare professionals, the public, and other relevant groups (eg, via publication, reporting in results databases, or other data sharing arrangements), including any publication restrictions***   The result of this study will be presented to Haramaya University research and community services affairs offices and will also be submitted to regional and national health bureaus. Moreover, it will be published on reputable journal to communicate with scientific societies. | | | | |
|  | | | 31b | ***Authorship eligibility guidelines and any intended use of professional writer***  **Authors:**  Tilayie Feto Gelano (BSc., MSc)^1^ ,Nega Assefa ( Ph.D., MPH., BSc )^2^, Yadeta [Dessie](http://haramaya.academia.edu/YadetaDessie) Bacha (Ph.D., MPH., BSc)^3^, Afendi Abdi Mahamed (MSc, BSc)^4^, Kedir Teji Roba (Ph.D., MPH., BSc)^5^,Mitiku Teshoma Hambisa (MPH., BSc)^6^  Feto T et al; License BioMed Central Ltd. This will be an open access article which will be distributed under the terms of the creative common attribution licence, which will permit unrestricted use, distribution and reproduction in any media, provided the original article will be properly cited. | | | | |
|  | | | 31c | - ***Plans, if any, for granting public access to the full protocol, participant-level dataset, and statistical code***   The study protocol will be submitted to the Haramaya university research office and Institutional Health Research Ethics Review After Publication. | | | | |
| Appendices | | |  |  | | | | |
| Informed consent materials | | | 32 | **Declaration of Voluntary Consent**  I have read/was read to me this consent form or participant information. I have clearly understood the purpose of the research, the procedure, risk and benefits, issue of confidentiality rights of participation and contact address for any queries. I have given the opportunity to ask questions for things that may have been unclear. I was informed that have the right to withdraw from the study at any time. Therefore, I declare that my voluntary consent to participate in this study with my signature.  Participant’s name and signature:_____ ____________ date___________  Investigator/s’ name and signature:_____________date ______________  NB: Participant’s information sheet and Informed consent is prepared separately for intervention and control group for clear communication of purpose of study and other concerns. | | | | |
| Biological specimens | | | 33 | - ***Plans for collection, laboratory evaluation, and storage of biological specimens for genetic or molecular analysis in the current trial and for future use in ancillary studies, if applicable***   In this study there is no plan to collect and store any specimen. The only sample which will be used is small drop of blood that will be obtained by lancet prick for fast anaemia test by hemaceu machine. | | | | |

*It is strongly recommended that this checklist be read in conjunction with the SPIRIT 2013 Explanation & Elaboration for important clarification on the items. Amendments to the protocol should be tracked and dated. The SPIRIT checklist is copyrighted by the SPIRIT Group under the Creative Commons “[Attribution-Non-commercial-NoDerivs 3.0 Unported.](http://www.creativecommons.org/licenses/by-nc-nd/3.0/)” license.

**References list**
